# Quality of life and hormone use: new validation results of MRS scale

**DOI:** 10.1186/1477-7525-4-32

**Published:** 2006-05-31

**Authors:** Jürgen Dinger, Thomas Zimmermann, Lothar AJ Heinemann, Diana Stoehr

**Affiliations:** 1 Center for Epidemiology & Health Research Berlin, Invalidenstr. 115, 10115 Berlin, Germany; 2 Jenapharm, Medical Affairs Gynecology, Otto-Schott-Str. 15, 07745 Jena, Germany; 3 University of Wuerzburg, Chair of Statistics, Am Hubland, 97074 Würzburg, Germany

## Abstract

**Background:**

The Menopause Rating Scale is a health-related Quality of Life scale developed in the early 1990s and step-by-step validated since then. Recently the MRS scale was validated as outcomes measure for hormone therapy. The suspicion however was expressed that the data were too optimistic due to methodological problems of the study. A new study became available to check how founded this suspicion was.

**Method:**

An open post-marketing study of 3282 women with pre- and post- treatment data of the self-administered version of the MRS scale was analyzed to evaluate the capacity of the scale to detect hormone treatment related effects with the MRS scale. The main results were then compared with the old study where the interview-based version of the MRS scale was used.

**Results:**

The hormone-therapy related improvement of complaints relative to the baseline score was about or less than 30% in total or domain scores, whereas it exceeded 30% improvement in the old study. Similarly, the relative improvement after therapy, stratified by the degree of severity at baseline, was lower in the new than in the old study, but had the same slope. Although we cannot exclude different treatment effects with the study method used, this supports our hypothesis that the individual MRS interviews performed by the physician biased the results towards over-estimation of the treatment effects. This hypothesis is underlined by the degree of concordance of physician's assessment and patient's perception of treatment success (MRS results): Sensitivity (correct prediction of the positive assessment by the treating physician) of the MRS and specificity (correct prediction of a negative assessment by the physician) were lower than the results obtained with the interview-based MRS scale in the previous publication.

**Conclusion:**

The study confirmed evidence for the capacity of the MRS scale to measure treatment effects on quality of life across the full range of severity of complaints before treatment. The difference of the relative improvement after therapy between the old and current study as well as the observed different sensitivity/specificity is – as a matter of probability – more likely to be caused by a bias introduced by the different application of the MRS scale than by real differences in the efficacy of the therapy. A randomized clinical trial would be needed to test the impact of the latter. The message for future studies is: The MRS scale should be only used as self-administered tool where the suggestive effect of questions raised by health professionals ("therapeutic optimism") can be largely excluded.

## Background

The Menopause Rating Scale (MRS) was developed in the early 1990s and later revised from a interview to self-administered symptom-profile instrument [1,2] to describe health-related quality of life (HRQoL).

The MRS scale became internationally well accepted as far as the usage in numerous countries is concerned. The first translation was from German into English [3]. Other translations followed [4] respecting international methodological recommendations. Currently, 23 language versions are available – either published or can be downloaded in PDF-format from the official website [4,5].

The validation of the revised MRS began some years ago [2,6-8] and led to quite acceptable psychometric characteristics of the scale [9]. Recently, we published in this journal data about the capacity of the scale to assess changes pre-/post hormone treatment. In absence of other data we analysed the MRS scale used in the old interview version and found astonishingly positive results for this type of validity. We discussed that this high consistency could be an overestimate due to the interference of the treatment-related changes perceived by patients and the physician as "interviewer" [10].

In the meantime, we got data for a new analysis based on the self-administered MRS, i.e. with no direct influence by the treating physician. It is the aim of this paper to present the new data and to discuss whether or not the conclusions about the sensitivity of the MRS as outcome measure were correct or need correction.

## Methods

The new study alike the previous one is an open post-marketing study. The study was conducted with a product for hormone therapy (Lafamme^® ^= 2 mg estradiol valerate + 2 mg dienogest) using the MRS scale as outcome measure under routine conditions of office-based gynecologists.

In brief, gynecologists from all parts of Germany participated in a post-marketing study on a voluntary basis. 4262 women who required hormone treatment started participation in this follow-up study after having prescribed the hormone therapy, but 117 women stopped soon. Beside other variables, the MRS scores were documented before therapy and 6 months after starting the hormone treatment.

The statistical analyses were performed with the commercial statistical package SAS 10.0.

## Results and comments

Altogether, 4145 women had baseline (pre-treatment) data, but only 3332 completed the MRS also after 6 months. Finally, 3282 women provided data with all necessary variables for analysis. Characteristics of these participants: The mean age (SD) was 53.9 (5.6) years (20% under 50 years, 66% between 50–60 years, and 14% over 60 years). The mean BMI was 26.1 (4.2).

The improvement of the health-related quality of life (HRQoL) – measured with the self-administered MRS scale – is described in Table 1. The means (SD) of the scoring points of the total scale (and the three subscales) improved significantly (p < 0.001 for all comparisons: operating analysis – Wilcoxon signed rank test) both in absolute and relative terms (= compared with baseline). In addition, this table compares these data with the relevant data from the old study [10] where the interview version of the MRS was used. In average, the scores improved by almost one third after six months of hormone treatment. However, the treatment effects were less pronounced than in the old study. The major difference between both studies is that the old one applied the MRS scale in an interview by the physician, in the new one however the MRS scale was self-administered and completed by the patient. The difference is notable for the psychological domain, but also for the total score.

**Table 1:** Improvement of MRS scores after therapy by absolute difference in scoring points.^§ ^Mean values (SD) for the total scale and for each subscale. Comparison between this study and the previous one (see text)

	Absolute change^§^		Relative change^# ^(%)	
	New study	Old study**	New study	Old study**
Total scale	8.2 (6.5)	9.3 (7.4)	30.7 (18.6)	36.1 (20.6)
Psychological subscale	2.9 (3.0)	3.8 (3.7)	27.8 (23.4)	34.5 (27.1)
Somatic subscale	3.6 (2.8)	3.6 (3.0)	33.5 (22.1)	37.3 (23.1)
Urogenital subscale	1.7 (2.0)	1.8 (2.3)	22.4 (24.3)	24.5 (25.3)

^§ ^Summary score "before therapy" minus "after therapy"

# Percent (%) change compared with the score before treatment: pre-treatment score minus post-treatment score divided by pre-treatment score multiplied by 100 (%)

** see Ref [9]

A possible bias between the initial study and this one could have been the difference in the drug formulation, but as a matter of probability this is likely to be explained by the form of application of the scale: The interview interferes obviously with the answer pattern of the patients, i.e. the patients might often intend to please the physician with a favorable assessment of his therapy. The pronounced difference in the psychological domain may support such an interpretation. Other notable differences between the two studies are the age of the study population and the type of HRT used. The participants of the first study were about 5 years younger on average, and another progestin with a different application regimen was used for treatment. We think, however, that the difference between the two MRS versions had a much bigger impact than the age or type of HRT. We assume that the effect of hormones did not play a role. This hypothesis however can only be tested with a randomized clinical trial that compares the efficacy of different products used in our two studies.

With the MRS scale various degrees of improvement ca be measured. This makes the scale suitable for follow-up of patients with few and mild complaints before therapy (= baseline) as well as those with severe symptomatology. This is presented in Table 2: The more severe the complaints were before treatment the better the effect regarding relative improvement of symptoms measured by the MRS, which speaks in favour of the clinical utility of the MRS as outcome measure. In comparison to the mean changes in all patients the differences between the self-administered and the interview version of the MRS were even more pronounced in all patients who had "mild", "moderate", or "severe" complaints at baseline. The differences between the test versions are only negligible in patients who had "no/little" complaints.

**Table 2:** Relative change of MRS scores as percent of the baseline score: Mean values (SD) of the relative change (= % improvement of the complaints) in four categories of severity at baseline. The range of baseline scores is given in brackets for each category of severity. New study is the current analysis, the old study was recently published: * see REF [9]

		**Severity of complaints at baseline**			
		No/little (0–4)	Mild (5–8)	Moderate (9–15)	Severe (16+)
Total score	New study	8.0 (12.9)	22.9 (12.9)	32.7 (13.9)	43.3 (15.5)
	Old study*	10.8 (10.6)	32.2 (9.8)	43.9 (11.8)	55.1 (13.8)
Psychological score	New study	5.8 (16.5)	18.2 (19.7)	28.1 (20.5)	42.8 (19.8)
	Old study*	6.0 (14.7)	27.6 (21.5)	43.7 (20.6)	57.1 (17.9)
Somatic score	New study	10.4 (20.4)	26.1 (20.8)	36.8 (17.8)	44.9 (17.3)
	Old study*	13.8 (17.3)	34.4 (18.5)	44.1 (16.9)	54.8 (15.9)
Urogenital score	New study	2.0 (18.1)	13.4 (19.0)	22.5 (22.3)	36.5 (22.0)
	Old study*	5.7 (13.9)	17.0 (20.6)	27.5 (23.6)	44.4 (22.6)

We also compared the MRS total score before and after hormone treatment with the norm values of MRS of the average female population aged 45–60 years [2,3]. This crude and simple comparison showed that the severely deteriorated distribution of complaints in the patient group before therapy – compared with the normal population – improved after therapy remarkably and reached almost the distribution of the normal population (data not shown).

In the previously published "old" study [10] we found a more exaggerated result. We discussed selection problems of the post-marketing study and also problems of the interview technique as reasons for an unexpected high proportion of patients without complaints after hormone therapy. This could support our hypothesis that a personal interview by the physician may bias the outcome toward over-estimation of the treatment effect.

Overall, it is worth noting that the MRS scale can obviously detect treatment effects even in persons with little or mild symptoms before therapy – although to a lesser degree. We cannot comment as to what extend the MRS scale is able to differentiate between true or placebo treatment effects. The study cannot contribute to such a discussion due to its study design. However, we consider a relative improvement of 20 to 30%, which represents for example the improvement from severe to moderate complaints, as clinically relevant difference. Therefore, we recommend using an improvement of more than 20% as threshold to establish an "effect" of a new treatment.

A last issue is the validity of outcome evaluation by means of the MRS scale when the subjective assessment by the physician is taken as "gold standard". The treating gynaecologist assessed the "success" of the treatment for each person. We compared the agreement between this judgement and the assessment derived from a defined cut-off point of the self-administered MRS total score.

In the previous study [10] we observed an unexpected good sensitivity/specificity: sensitivity (correct prediction of a positive assessment by the physician) 70.8% and specificity (correct prediction of a negative assessment by the physician) 73.5%. We expressed concern that the degree of concordance might be overestimated due to the above mentioned study limitation (interview by physician). Our current study based directly on patients' information (physician neither present when the scale was completed nor when the scale was evaluated) came to somewhat but not much lower conformity with physicians' assessment. Table 3 shows that with a cut-off point between 20 and 22% of therapy-related improvement the sensitivity is around 70%, but the specificity is only between 50–60% and thereby markedly lower than in the previous study. In the previous study, the clinical judgement about "treatment success" was done by the physician who was responsible for the chosen treatment. We assume that in many cases the true treatment effect is better reflected by the self-administered MRS then by this form of clinical judgement. Together with the above discussed relative improvement of the complaints after therapy, the sensitivity and specificity results obtained in our current study point into the direction of a bias mainly introduced by application of the MRS scale as interview by the treating physician.

**Table 3:** Values for Sensitivity and Specificity of predicting physicians assessment of "successful therapy". Sensitivity and specificity is listed for a series of cut-off points (given in percent of baseline total score) for relative score improvement on the total MRS-scale.

Cut-off Point Relative score improvement (%)	Sensitivity (%)	Specificity (%)
≥ 5	92.1	22.7
≥ 10	87.4	33.6
≥ 15	81.6	42.8
≥ 20	73.4	53.3
≥ 22	70.1	57.2
≥ 25	63.9	62.9
≥ 30	54.6	75.6
≥ 35	44.1	79.9
≥ 40	33.1	88.2
≥ 45	24.2	91.3
≥ 50	14.7	94.3

We hope to get access to data of double-blinded, randomized clinical trials to confirm the results of this validation study, i.e. to know the impact of treatment efficacy. But even on the basis of the information available from our two studies, the available information is re-assuring concerning methodological quality of the MRS scale as clinical utility.

## Conclusion

The study confirmed evidence for the capacity of the MRS scale to measure treatment effects on quality of life across the full range of severity of complaints before treatment. The difference of the relative improvement after therapy between the old and current study as well as the observed different sensitivity/specificity is – as a matter of probability – more likely to be caused by a bias introduced by the different application of the MRS scale than by real differences in the efficacy of the therapy. A randomized clinical trial would be needed to test the impact of the latter. The message for future studies is: The MRS scale should be only used as self-administered tool where the suggestive effect of questions raised by health professionals ("therapeutic optimism") can be largely excluded.

## Competing interests

**TZ **is employee of a company that produces hormone products.

The authors, however, see no conflict of interest as far as the validation of the MRS scale is concerned.

## Authors' contributions

**JD: **contributed to the design of the study, the validation analysis and the writing of the manuscript; **TZ: **responsible for design and execution of the post-marketing study; **LAJH**: one of the developers of the MRS scale, responsible for the design of this validation analysis, and involved in writing the manuscript; **DS**: responsible for setting up and managing the database for different analyses, and for running all analyses.
